# Persistence of an outbreak of gonorrhoea with high-level resistance to azithromycin in England, November 2014‒May 2018

**DOI:** 10.2807/1560-7917.ES.2018.23.23.1800287

**Published:** 2018-06-07

**Authors:** Christa Smolarchuk, Adrian Wensley, Simon Padfield, Helen Fifer, Andrew Lee, Gwenda Hughes

**Affiliations:** 1 HIV & STI Department, Public Health England, Colindale, London, United Kingdom; 2 Field Epidemiology Service, Public Health England, Leeds, United Kingdom; 3 These authors contributed equally to this work and share first authorship; 4 Bacteriology Reference Department, National Infection Service, Public Health England, Colindale, London, United Kingdom; 5 Public Health England, Yorkshire and Humber, Leeds

**Keywords:** gonorrhoea, Neisseria gonorrhoeae, antimicrobial resistance, England, azithromycin, STI, MSM: men who have sex with men

## Abstract

Between November 2014 and May 2018, 118 laboratory-confirmed cases of high-level azithromycin resistant *Neisseria gonorrhoeae* were identified in England. Cases emerged among heterosexuals in Leeds but spread across England and into sexual networks of men who have sex with men as the outbreak progressed. The few epidemiological links identified indicate substantial under-diagnosis of cases and this, along with the upturn in cases in 2017, highlights the difficulties in controlling the outbreak.

An outbreak of high-level azithromycin resistant *Neisseria gonorrhoeae* (HL-AziR; minimum inhibitory concentration (MIC) ≥ 256 mg/L) was first identified in Leeds in the north of England in 2015, where a local incident control team was convened [1-3]. This transitioned into a national response in early 2016 as increasing numbers of cases were identified in other parts of the country. The outbreak is still ongoing, despite a number of interventions, including increasing awareness among clinicians and microbiologists, a national resistance alert issued by Public Health England (PHE) [4], and intensive follow-up of cases and their partners through partner notification (PN) by sexual health services. This report provides an update on the epidemiology of HL-AziR cases in England and describes the progression of the outbreak up to May 2018.

## Description of the outbreak

**Figure 1:**
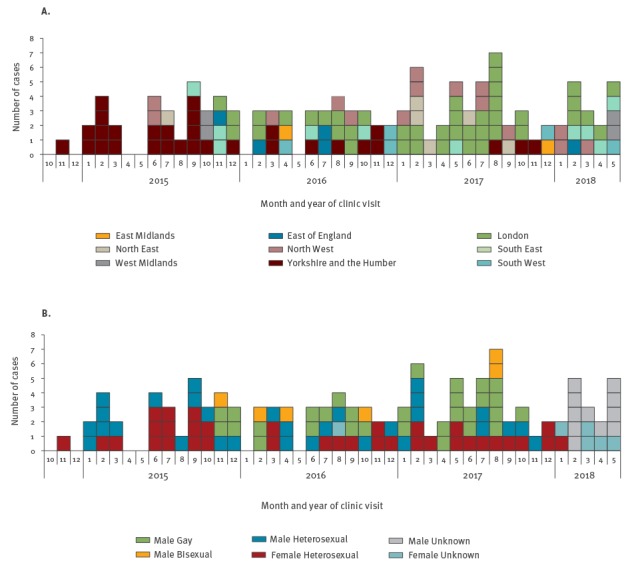
Confirmed cases of high-level azithromycin resistant *Neisseria gonorrhoeae* by (A) area of residence^a^ and (B) sexual orientation, England, November 2014 and May 2018 (n=118)

Between November 2014 and May 2018, 118 laboratory confirmed cases of HL-AziR were identified in England. Despite concerns of rapid escalation and spread, the average number of new cases identified per month remained relatively stable initially, but cases per month increased from 2.6 in 2015 to 3.3 in 2017 and 3.4 in 2018 (Figure 1). There was one reported treatment failure in early 2018, but this infection was likely acquired outside of the United Kingdom (UK) [5,6]. This case was also the first global report of an isolate that had HL-AziR and was also resistant to ceftriaxone.

Data for end of May 2018 could be incomplete as there is a lag time of 2 to 3 weeks in antimicrobial susceptibility testing.

^a^ Public Health England Centre.

The outbreak emerged at the end of 2014 among young heterosexuals (median age 18 years, range 16‒53; n = 15) living in deprived areas in Leeds. No specific risk factors, such as common educational establishments, use of online dating applications (apps), or venues, were identified in the initial investigation. Most heterosexual cases were positive at a single site of infection (46/71), and were predominantly genital infections (41/50), among those where site of infection was known (Table 1). Re-attendance for test of cure among these cases was relatively low (10/15), even with intensive follow-up of cases, and clinicians reported difficulty in identifying partners.

**Table 1:** Characteristics and demographics of laboratory-confirmed cases of high-level azithromycin resistant *Neisseria gonorrhoeae*, England November 2014 ‒May 2018

Patient characteristics		Women	Heterosexual Men	MSM	Men unknown	Total
		n (col %)				
Total cases (n)		41 (100)	30 (100)	36 (100)	11 (100)	118 (100)
Ethnicity						
	**White**	**31 (76)**	**19 (63)**	**24 (67)**	**0 (0)**	**74 (63)**
	**Black**	**0 (0)**	**5 (17)**	**6 (17)**	**0 (0)**	**11 (9)**
	**Other**	**2 (5)**	**5 (17)**	**6 (17)**	**0 (0)**	**13 (11)**
	**Unknown**	**8 (20)**	**1 (3)**	**0 (0)**	**11 (100)**	**20 (17)**
Age group (years)						
	**15–19**	**21 (51)**	**9 (30)**	**4 (11)**	**1 (9)**	**35 (30)**
	**20–24**	**14 (34)**	**8 (27)**	**6 (17)**	**2 (18)**	**30 (25)**
	**25–34**	**5 (12)**	**8 (27)**	**14 (39)**	**5 (45)**	**32 (27)**
	**≥ 35**	**1 (2)**	**5 (17)**	**12 (33)**	**3 (27)**	**21 (18)**
	**Yes**	**28 (68)**	**24 (80)**	**24 (67)**	**0 (0)**	**76 (64)**
	**No**	**2 (5)**	**3 10()**	**11 (31)**	**0 (0)**	**16 (14)**
	**Unknown**	**11 (27)**	**3 (10)**	**1 (3)**	**11 (100)**	**26 (22)**
World region of birth						
	**UK**	**28 (72)**	**24 (80)**	**24 (67)**	**0 (0)**	**76 (68)**
	**Other**	**2 (5)**	**3 (10)**	**11 (31)**	**0 (0)**	**16 (14)**
	**Unknown**	**9 (23)**	**3 (10)**	**1 (3)**	**6 (100)**	**19 (17)**
HIV status						
	**Positive**	**0 (0)**	**0 (0)**	**7 (19)**	**0 (0)**	**7 (6)**
	**Negative/Undiagnosed**	**27 (66)**	**25 (83)**	**27 (75)**	**0 (0)**	**79 (67)**
	**Unknown**	**14 (34)**	**5 (17)**	**2 (6)**	**11 (0)**	**32 (27)**
Index of multiple deprivation						
	**Most deprived**	**13 (32)**	**11 (37)**	**7 (19)**	**0 (0)**	**31 (26)**
	**2nd most deprived**	**5 (12)**	**2 (7)**	**16 (44)**	**0 (0)**	**23 (19)**
	**3rd most deprived**	**2 (5)**	**2 (7)**	**7 (19)**	**0 (0)**	**11 (9)**
	**4th most deprived**	**2 (5)**	**5 (17)**	**3 (8)**	**0 (0)**	**10 (8)**
	**Least deprived**	**4 (10)**	**3 (10)**	**1 (3)**	**0 (0)**	**8 (7)**
	**Unknown**	**15 (37)**	**7 (23)**	**2 (6)**	**11 (100)**	**35 (30)**
Enhanced data on cases (n)^a^		35 (100)	30 (100)	36 (100)	0 (100)	101 (100)
Symptoms^b^						
	**Discharge and/or Dysuria**	**13 (37)**	**25 (83)**	**19 (53)**	**0**	**57 (56)**
	**No Discharge and/or Dysuria**	**18 (51)**	**4 (13)**	**15 (42)**	**0**	**37 (37)**
	**Unknown**	**4 (11)**	**1 (3)**	**2 (6)**	**0**	**7 (7)**
Site(s) positive^b,c^						
	**Genital**	**21 (60)**	**25 (83)**	**9 (25)**	**0**	**55 (54)**
	**Rectal**	**5 (14)**	**0 (0)**	**8 (22)**	**0**	**13 (13)**
	**Throat**	**7 (20)**	**1 (3)**	**13 (36)**	**0**	**21 (21)**
	**Other**	**1 (3)**	**1(3)**	**0 (0)**	**0**	**2 (2)**
	**Unknown sites**	**11(31)**	**4 (13)**	**16 (44)**	**0**	**31 (31)**
Multiple/single site(s) ^b,c^						
	**Single site**	**16 (46)**	**25 (83)**	**12 (33)**	**0**	**53 (53)**
	**Multiple sites**	**8 (23)**	**1 (3)**	**8 (22)**	**0**	**17 (17)**
	**Unknown sites**	**11(31)**	**4 (13)**	**16 (44)**	**0**	**31 (31)**
Test of cure						
	**Yes**	**20 (57)**	**18 (60)**	**31 (86)**	**0**	**69 (68)**
	**No**	**11 (31)**	**11 (37)**	**4 (11)**	**0**	**26 (26)**
	**Unknown**	**4 (11)**	**1 (3)**	**1 (3)**	**0**	**6 (6)**
Partners (past 3 months)						
	**0–1**	**14 (40)**	**12 (40)**	**6 (17)**	**0**	**32 (32)**
	**2–5**	**11 (31)**	**14 (47)**	**16 (44)**	**0**	**41 (40)**
	**6–10**	**0 (0)**	**0 (0)**	**6 (17)**	**0**	**6 (6)**
	**11 +**	**0 (0)**	**1 (3)**	**3 (8)**	**0**	**4 (4)**
	**Unknown**	**10 (29)**	**3 (10)**	**5 (14)**	**0**	**18 (18)**
Venues (past month)^d^						
	**Yes**	**0 (0)**	**0 (0)**	**3 (8)**	**0**	**3 (3)**
	**No**	**0 (0)**	**0 (0)**	**1 (3)**	**0**	**1 (1)**
	**Unknown**	**35 (100)**	**30 (100)**	**32 (89)**	**0**	**97 (96)**
Websites (past month)						
	**Yes**	**0 (0)**	**0 (0)**	**2 (6)**	**0**	**2 (2)**
	**No**	**0 (0)**	**0 (0)**	**3 (8)**	**0**	**3 (3)**
	**Unknown**	**35 (100)**	**30 (100)**	**31 (86)**	**0**	**96 (95)**

Source: GUMCAD STI Surveillance System and enhanced surveillance of cases. STI: sexually transmitted infection; UK: United Kingdom.

^a^ Data from enhanced surveillance on all cases up to March 2017 only (n = 71).

^b ^Abbreviated enhanced surveillance only for cases April to December 2017 (n = 30).

^c ^Patients may not have been tested for all sites.

^d ^Refers to sex on premises, venues including saunas and sex clubs.

Cases spread geographically (Figure 1A) and into sexual networks of men who have sex with men (MSM) (Figure 1B) as the outbreak progressed. Cases among MSM were first identified in November 2015 mainly from a large, high-throughput clinic in London, which serves a substantial MSM population. Of the 36 cases identified among MSM, seven were known to be HIV-positive (Table 1). MSM cases were generally older than heterosexual cases (median age 30 years, range 17‒65) but also lived in deprived areas. Only a few MSM reported using apps or websites to meet sex partners, but this might be under-reported. Among MSM, most had a single site of infection (12/20), but the most common site of infection was pharyngeal (13/20) (Table 1). Re-attendance for test of cure was higher among MSM (31/36) compared with heterosexuals.

## Sexual partnerships and transmission networks

**Figure 2:**
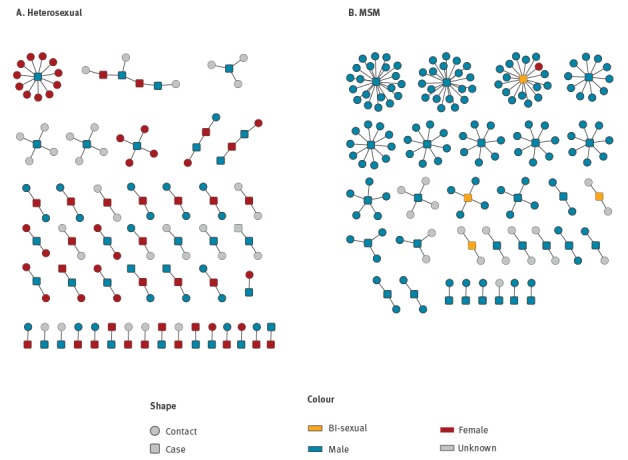
Partner network diagram of laboratory confirmed cases of high-level azithromycin resistant *Neisseria gonorrhoeae* and their reported partners by (A) heterosexual and (B) men who have sex with men (MSM) cases, England, November 2014 to December 2017 (n = 324)

Six cases among MSM also exhibited bisexual behaviour suggesting a degree of fluidity between sexual networks, which may have facilitated more rapid and widespread transmission. Partner information was reported through enhanced surveillance for 82% (83/101) of cases up to December 2017 (52 heterosexual and 31 MSM). The median number of partners in the previous 3 months reported per case was two among MSM (range 1‒20 partners) and 1.5 among heterosexuals (range 1‒11 partners). There were very few direct links identified between heterosexual cases (Figure 2A) and no direct links identified between any MSM cases (Figure 2B). Among 52 heterosexual cases, 25 were epidemiologically linked to another case establishing 11 separate transmission networks (the largest linking four early Leeds cases).

Black lines connecting shapes indicate cases and/or partners with known epidemiological links.

## Partner notification and case ascertainment

Effective PN is essential to controlling STI-related outbreaks, but despite best efforts by clinic staff, success was limited. Only 35% (33/95) of partners reported by heterosexual cases and 0% (0/153) of partners reported by MSM cases were verified by sexual health services as successfully contacted and tested (Table 2). Among the partners who were tested, 14 of 29 were confirmed as new cases. Most of the reported partners were uncontactable because contact details were not disclosed by cases or were unavailable for casual partners. In the absence of effective PN, high frequency of asymptomatic infection is likely to further contribute to sustained transmission; up to December 2017, 15 of 36 MSM, 4 of 30 heterosexual men, and 18 of 35 women were asymptomatic (Table 1). Among the four linked heterosexual cases in Leeds, one became re-infected, further highlighting the potential impact of untreated partners on spread of the outbreak.

**Table 2:** Outcomes of partners notified by high-level azithromycin resistant *Neisseria gonorrhoeae* (HL-AziR) cases verified by sexual health clinics, England, November 2014‒December 2017 (n=83)

Partner notification	Heterosexual	Men who have sex with men	Total
	n/N	n/N	n/N
Partners			
Reported by cases	**95/95**	**153/153**	**248/248**
Partner notification			
Successfully contacted/verified by sexual health clinic	**33/95**	**0/153**	**33/248**
Gonorrhoea testing			
Tested	**29/33**	**0**	**29/33**
Not tested/Unknown	**4/33**	**0**	**4/33**
Test result			
Gonorrhoea positive	**21/29**	**0**	**21/29**
Gonorrhoea negative	**8/29**	**0**	**8/29**
Partners who became cases			
Confirmed case	**14/21**	**0**	**14/21**
Probable case a	**3/21**	**0**	**3/21**
Non-cases b (gonorrhoea positive; sensitive to azithromycin)	**4/21**	**0**	**4/21**

^a^ Gonorrhoea-positive, partner of a HL-AziR case, no azithromycin sensitivity available.

^b^ Gonorrhoea-positive, isolate sensitive to azithromycin.

All cases were confirmed as high-level azithromycin resistant by PHE’s national reference laboratory. An estimated 63% of all gonorrhoea diagnosed in England are cultured for antimicrobial susceptibility testing [7]. On multiple occasions, PHE attempted to improve the rate of referral of gonococcal isolates with suspected resistance, identified by primary diagnostic laboratories to PHE’s national reference laboratory for confirmatory antimicrobial susceptibility testing. Referrals of gonococcal isolates with suspected azithromycin resistance increased from 34% (96/282) to 50% (219/437) and gonococcal isolates with suspected ceftriaxone resistance increased from 27/89 isolates to 14/41 in 2015 and 2017 (up to October), respectively, but remained sub-optimal overall [8]. Issues related to low referrals rates by primary diagnostic laboratories included a lack of standard processes and staff awareness to ensure that isolates were referred, often exacerbated by high staff turn-over and difficulty with retrieval of cultures for transport to the national reference laboratory.

## Discussion and conclusions

There has been sustained transmission of HL-AziR in England since November 2014. Few cases had identified epidemiological links and this, along with the limited effectiveness of PN, suggests there is likely a considerable reservoir of undiagnosed infection fuelling transmission. Despite the lack of direct links identified between most cases, the early geographic clustering, and multiple bisexual cases potentially linking heterosexual and MSM sexual networks, strongly suggested these cases belonged to a discrete outbreak, which was confirmed by whole genome sequencing (WGS) [9]. WGS of isolates up to February 2017 demonstrated that most of the cases shared a distant common ancestor, and 37 of 60 cases were genetically similar (clonal) [9].

The HL-AziR clonal spread among initial outbreak cases in Leeds [9] may have been facilitated by an identified sexual network including four cases, one of whom also became re-infected and indicated ongoing transmission over several months. A local outbreak control team was convened and the last case was identified in October 2015. A brief resurgence of cases in Leeds in 2016 (one case diagnosed in June and then three cases in October and November) were not identified as having epidemiological links to the initial outbreak cases. WGS demonstrated that the isolates from the later cases had evolved, but were still closely related, genetically [9]. This suggests that even with the decline in cases in Leeds, the strain continued to circulate.

Gonorrhoea treatment guidelines published by the World Health Organisation (WHO), the UK, Europe and many other countries recommend first-line dual therapy with ceftriaxone and azithromycin to treat gonorrhoea [10,11] in an attempt to delay the development of resistance to ceftriaxone, which despite sporadic reports, remains rare [12-17]. While low-level azithromycin resistance (0.5 mg/L < MIC < 256 mg/L) in gonorrhoea is increasing in many countries [18], HL-AziR cases have previously been uncommon or have belonged to small contained outbreaks [19,20], suggesting that HL-AziR may lead to a fitness cost. The data presented here tend not to support this view, although information on population-wide transmission dynamics of different gonococcal strains is sparse [21].

The outbreak received substantial national and local media attention, and multiple communications aimed at providing advice and improving awareness among primary diagnostic laboratories and sexual health services were undertaken. However, the limited effectiveness of PN and poor re-attendance for test of cure, even with intense follow-up, severely hampered control efforts. Referral of suspected azithromycin-resistant gonococcal isolates by primary diagnostic laboratories for confirmatory testing by PHE’s national reference laboratory remains sub-optimal.

The increase in HL-AziR cases during 2017, predominantly in MSM in London, demonstrates how an outbreak of gonorrhoea can spread geographically, and within and between sexual networks, even with intensive interventions in place. The outbreak highlights the potential for rapid transmission of highly resistant or even untreatable strains of gonorrhoea in the future. Research to identify effective approaches to encourage and empower people to get tested for STIs and to identify barriers to accessing healthcare are urgently required in order to implement unified, enhanced strategies in the prevention of gonorrhoea.
